# Practices and attitudes of Thai community pharmacists in managing diabetic peripheral neuropathy using neurotropic B vitamins

**DOI:** 10.1080/20523211.2026.2701910

**Published:** 2026-07-20

**Authors:** Kitiyot Yotsombut, Thanompong Sathienluckana, Sirinoot Palapinyo, Naeti Suksomboon

**Affiliations:** a Faculty of Pharmaceutical Sciences, Chulalongkorn University, Bangkok, Thailand; b Faculty of Pharmacy, Siam University, Bangkok, Thailand; c Faculty of Pharmacy, Mahidol University, Bangkok, Thailand

**Keywords:** Pharmacist, diabetic peripheral neuropathy, neurotropic B vitamins, vitamin B1, vitamin B6, vitamin B12

## Abstract

**Background:**

Diabetic peripheral neuropathy (DPN) affects approximately half of individuals with diabetes. Community pharmacists play a pivotal role in the early management of DPN. This exploratory study examined community pharmacist practices in DPN assessment and management, with particular focus on attitudes toward and factors influencing neurotropic B vitamin recommendations.

**Methods:**

A cross-sectional survey was conducted among a convenience sample of community pharmacists in Bangkok, Thailand. The questionnaire focused on pharmacists’ attitudes and practices in managing patients presenting with negative symptoms, positive symptoms, or diagnosed DPN.

**Results:**

Ninety community pharmacists participated in this study. Most patients presented with mild symptoms. Symptom severity was primarily assessed based on patient history and pain scales. Among patients presenting with negative symptoms and overt DPN, the most frequently recommended medication was a combined neurotropic B vitamin (48.11% and 44.73%, respectively). The factor associated with likelihood of recommending combined neurotropic B vitamins was reasonable product price, while guideline recommendation, package labelling, and brand reputation showed inverse association with recommendation behaviour.

**Conclusion:**

Community pharmacists frequently recommend neurotropic B vitamins for early-stage and painless DPN – presentations not addressed in current clinical guidelines. The inverse associations between guideline endorsement and recommendation behaviour likely reflect these guideline gaps rather than inappropriate practice. Pharmacy-specific clinical guidelines and clearer product labelling addressing both painful and painless DPN are needed. Given the exploratory design and convenience sampling from Bangkok, findings should be interpreted as hypothesis-generating.

## Background

The global prevalence of diabetes mellitus (DM) is projected to reach 853 million individuals by the year 2050 (International Diabetes Federation, 2024). In Thailand, DM was the third leading cause of death in 2022, accounting for 9.77% of all deaths (Health Information System Development Office, 2022). The disease leads to a range of macrovascular and microvascular complications including diabetic nephropathy, neuropathy and retinopathy (Fowler, 2008). Diabetic peripheral neuropathy (DPN) is one of the most common microvascular complications affecting 50% of people with diabetes (Fowler, 2008; Pop-Busui et al., 2022). The main symptoms include positive symptoms such as burning pain, tingling, and over-sensitized sensations, as well as negative symptoms like numbness, loss of sensation and balance impairment (Inoue et al., 2016; Nold & Nozaki, 2020). These can lead to severe consequences such as foot ulcers, infections, and even amputations, substantially impairing patients’ quality of life when early treatment is neglected.

Community pharmacists play a vital role in primary care by providing accessible early management of DPN. Understanding current attitudes and practices is essential for identifying knowledge gaps and informing the development of educational strategies and practice standards for pharmacists in nation. Among pharmacological treatment, neurotropic B vitamins, comprising combinations of vitamins B1 (thiamine), B6 (pyridoxine), and B12 (cobalamin), have been widely used due to their neuroprotective and nerve repair properties (Baltrusch, 2021). The body of evidence suggests that these vitamins can alleviate neuropathic symptoms and improve nerve function (Liew et al., 2019; Rayner et al., 2025). Owing to the availability as over-the-counter (OTC) drug and favourable safety profile, combined neurotropic B vitamins are commonly recommended in community pharmacy practice (Sathienluckana et al., 2024; Schellack et al., 2025).

Despite growing evidence supporting the roles of neurotropic B vitamins in nerve function, their integration into routine DPN management remains poorly characterised, including in community pharmacy settings. Existing clinical guidelines primarily focus on painful DPN and offer limited guidance for pharmacists managing early or painless neuropathic symptoms. Furthermore, little is known about the real-world factors influencing pharmacists’ recommendation of neurotropic B vitamins. Addressing these gaps is essential for developing pharmacist-specific guidance and optimising early DPN care.

This exploratory, cross-sectional survey examined Thai community pharmacists’ perceptions and practices regarding the management of DPN, with a particular focus on neurotropic B vitamin use, to identify opportunities for enhancing pharmacist-led care in primary care setting.

## Method

This study was conducted and reported in accordance with the Strengthening the Reporting of Observational Studies in Epidemiology (STROBE) guidelines for cross-sectional studies. The study protocol received approval from the Ethics Committee of COA. No. 260/68, Chulalongkorn University. All respondents provided informed consent to participate in the study.

**Figure 1.**
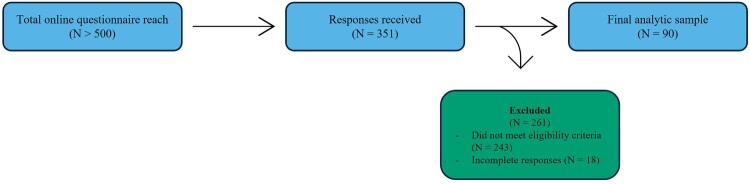
Schematic diagram of online respondents’ flow.

The cross-sectional questionnaire survey was conducted among community pharmacists practicing in Bangkok, Thailand. The required sample size was determined using a finite population correction method, assuming a 95% confidence level, 5% margin of error, and an estimated population proportion (p) of 0.5. There were 3,429 pharmacies in Bangkok registered with the Food and Drug Administration, Thailand, in 2024. Therefore, the required sample size was 345. This study used a convenience sampling approach (Figure 1). The self-administered questionnaire link was disseminated via online social networking platforms commonly used by community pharmacists, with a reach of over 500 potential respondents. Respondents were screened for eligibility based on the following criteria: (1) active practice in Bangkok area and (2) experience with at least one patient presenting symptoms such as numbness or loss of sensation in the hands or feet, burning pain, tingling, lancinating pain, pins and needles, or a diagnosis of DPN in the last 3 months to reduce recall biases. A total of 351 responses were received; 261 were excluded due to ineligibility or incomplete responses, resulting in 90 pharmacists included in the final analysis.

The questionnaire used in this study was developed by the research team based on the Clinical Guidance for Neuropathic Pain 2020 issued by the Thai Association for the Study of Pain (Thai Association for the Study of Pain, 2020). Its content validity was evaluated by three pharmacist experts with experience in pharmaceutical care, including the diagnosis, evaluation, and management of diabetic peripheral neuropathy (DPN). All experts hold board certification in pharmacotherapy, with specialisation in neuropsychiatry, endocrinology, and community pharmacy, respectively, as accredited by the Pharmacy Council of Thailand. Each serve as a full-time lecturer in the Faculty of Pharmacy.

The questionnaire consisted of two parts: a screening part and a main section. The main section collected data on respondent demographics, patient assessment practices, referral behaviours, perceptions of DPN, treatment patterns, and behaviours regarding the recommendation of neurotropic B vitamins. These data, including the use of standardised neuropathic pain screening tools, such as the Douleur Neuropathique 4 (DN4) questionnaire, were assessed based on pharmacists’ self-reported practices. No verification of actual DN4 administration or documentation in routine practice was performed. It also explored perceptions of community pharmacists’ role and barriers to recommending neurotropic B vitamins using a three-level Likert type rating scale. Based on expert assessments, the index of item-objective congruence ranged from 0.67–1.00, indicating satisfactory content validity. Subsequently, the questionnaire was pilot tested with 30 Thai community pharmacists to assess clarity and usability. Cronbach’s alpha coefficients for the knowledge and attitude sections were 0.78 and 0.83, respectively, demonstrating acceptable internal consistency of the instrument.

Descriptive statistics were used to summarise pharmacists’ responses. Multivariable logistic regression analyses were conducted to explore associations between selected product-related factors and pharmacists’ likelihood of recommending combined neurotropic B vitamins for patients presenting with negative symptoms, positive symptoms, or overt diabetic peripheral neuropathy. For analytic purposes, a higher likelihood of recommending was operationally defined as a self-reported probability of ≥60%. This threshold was selected pragmatically to distinguish relatively higher versus lower likelihood of recommending and should be interpreted as exploratory rather than clinically definitive.

Given the limited sample size, regression analyses were performed to generate hypotheses regarding potential associations rather than to establish predictive or causal relationships. Multicollinearity among independent variables was assessed using variance inflation factors (VIFs), with values <10 indicating acceptable collinearity. Odds ratios (ORs) with 95% confidence intervals (CIs) were reported to describe the direction and magnitude of associations. Statistical significance was defined as a two-tailed *p*-value <0.05. All analyses were conducted using SPSS version 29.0.2 (IBM Corp., Armonk, NY).

## Results

### Demographic and baseline characteristics

A total of 90 community pharmacists in Bangkok completed the questionnaire during the study period. Most respondents were female (68%), while 32% were male. The mean age was 36.64 years (SD = 7.84). Respondents reported an average of 9.76 years (SD = 5.27) of experience in community pharmacy practice, and 90% had more than six years of professional experience. The distribution of workplace settings was evenly split between independent and chain pharmacies. Regarding the accreditation status of drug stores, 57% of the pharmacies were classified as Standard Drug Stores, while 43% held Good Pharmacy Practice (GPP) accreditation. Notably, GPP accreditation was significantly more common among independent pharmacies (73%) compared to chain pharmacies (*p* < 0.05) (Table 1).

**Table 1.** Pharmacists’ demographic and clinical practice characteristics.

Characteristics		Overall (n = 90)
**Demographics**		
Gender, n (%)	Male	29 (32%)
	Female	61 (68%)
Age, n (%)	25–34 years	41 (46%)
	35–44 years	42 (47%)
	45–54 years	4 (4%)
	≥ 55 years	3 (3%)
Highest educational background, n (%)	BPharm	49 (54%)
	PharmD	40 (44%)
	Postgraduate (Master, PhD or Residency)	1 (1%)
**Clinical practice characteristics**		
Year of experience in community pharmacy practice, n (%)	1–5 years	9 (10%)
	6–10 years	60 (67%)
	11–20 years	19 (21%)
	>21 years	2 (2%)
Type of drug store, n (%)	Independent store without branches	41 (46%)
	Independent store with branches	4 (4%)
	Chain store	45 (50%)
Type of drug store accreditation, n (%)	GPP	39 (43%)
	Standard drug store	51 (57%)
Role in drug store, n (%)	Owner	26 (29%)
	Manager	1 (1%)
	Staff member or person in charge	63 (70%)

BPharm: Bachelor of Pharmacy, GPP: Good Pharmacy Practice, PharmD: Doctor of Pharmacy, PhD: Doctor of Philosophy

### Attitudes and practices in symptom assessment, medical referral and treatment

Pharmacists’ practices were examined in patient assessment, medical referral and treatment recommendations related to (1) ‘negative symptoms’ including numbness or loss of sensation, (2) ‘positive symptoms’ including burning pain, tingling, lancinating and pin and needles at hands or feet, and (3) overt DPN. These classifications were employed to capture the full range of symptom presentations, particularly since the pilot study revealed that some respondents were unfamiliar with the clinical term ‘diabetic peripheral neuropathy,’ although they recognised its symptom patterns commonly observed in patients with diabetes.

Symptom severity was most commonly assessed through history taking/interviewing (97–100%), patient-reported information or previous diagnosis (57–74%), and pain scales (60–76%). In contrast, the reported use of standardised screening tools for neuropathic pain, such as the Douleur Neuropathique 4 (DN4) questionnaire (Thai Association for the Study of Pain, 2020), was relatively uncommon, with usage rates ranging from 11% to 24% (Table 2). Across all symptom types, approximately half of the patients visiting pharmacies were reported to have mild symptom severity: 52.54% for negative symptoms, 50.59% for positive symptoms, and 48.13% for overt DPN. Patients with moderate or severe symptoms were more likely to be referred to a physician, with referral rates of 32.34–46.61% for moderate symptoms and 72.13–79.53% for severe symptoms, depending on symptom classification.

**Table 2.** Pharmacist practices in patient assessment, medical referral and treatment recommendations.

Characteristics		Negative symptoms	Positive symptoms	Overt DPN
Severity assessment method, n (% of respondents)	History taking/ interviewing*	90 (100%)	87 (99%)	85 (97%)
	Previous diagnosis**	51 (57%)	59 (67%)	65 (74%)
	Pain scale	56 (62%)	67 (76%)	53 (60%)
	DN4	10 (11%)	14 (16%)	21 (24%)
	Other specify	2 (2%)	6 (7%)	7 (8%)
Severity level distribution, % of encountered patients	Mild level	52.54	50.59	48.13
	Moderate level	34.72	34.14	33.49
	Severe level	10.01	12.33	11.30
	Not known	3.02	2.94	7.09
Medical referral rate, % of encountered patients	Mild level	8.74	13.80	24.82
	Moderate level	32.34	41.83	46.61
	Severe level	79.53	73.18	72.13
	Not known	19.26	20.48	21.90
Treatment approach, % of encountered patients	Initiation with medicines, vitamins or supplements	56.92	49.22	40.78
	Refill or repeat medicines, vitamins or supplements	36.44	43.80	52.22
	Provide only non-pharmacological advice	6.63	6.99	7.00
Recommended medication, % of encountered patients	Neurotropic B vitamin (vitamin B1, B6 and B12)	48.11	34.47	44.73
	Vitamin B12	31.89	27.23	32.35
	Gabapentin/ pregabalin	19.76	39.98	24.68
	Other Vitamin / Mineral	5.22	3.55	4.20
	Oral nonsteroidal anti-inflammatory drug	5.73	9.09	4.56
	Tricyclic antidepressants/ serotonin-norepinephrine reuptake inhibitors	2.47	3.74	2.31
	Topical cream for pain relief	2.98	2.45	1.94
	Alfa-lipoic acid	1.16	1.34	2.80
	Paracetamol	1.24	1.30	1.14
	Others, please specify	0.72	1.08	1.02

* based on duration and progression of symptoms.

** based on patient-reported information or previous diagnosis by physicians.

DN4: Douleur Neuropathique 4, DPN: diabetic peripheral neuropathy.

The treatment approach provided to the patients primarily was initiation with medications, vitamins or supplements (40.78–56.92%) and refill or repeat medications (36.44–52.22%). In contrast, non-pharmacological counselling alone was infrequently provided, with rates ranging from 6.63% to 7.00% (Table 2).

Among patients presenting with negative symptoms or overt DPN who were receiving medications, the three most commonly recommended medications were combined neurotropic B vitamins (48.11% and 44.73%), single vitamin B12 (31.89% and 32.35%) and gabapentin or pregabalin (19.76% and 24.68%), respectively. Patients with positive symptoms were most likely to receive gabapentin or pregabalin (39.98%), followed by combined neurotropic B vitamins (34.47%) and single vitamin B12 (27.23%) (Table 2).

### Practices in neurotropic B vitamins for DPN management

**Figure 2.**
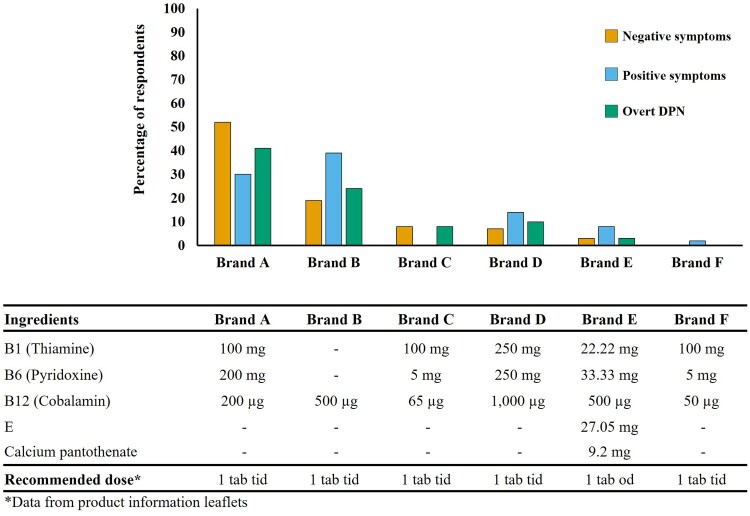
Preferred neurotropic B vitamin formulations recommended by community pharmacists according to symptom presentation. The figure illustrates community pharmacists’ preferred neurotropic B vitamin products and formulations (amount/tablet) for the management of diabetic peripheral neuropathy (DPN), stratified by symptom presentation. Percentages represent the proportion of pharmacists reporting each product preference. DPN: diabetic peripheral neuropathy, od = omne in die (once a day), tab = tablet, tid = ter in die (three times a day)

A combined neurotropic B vitamin formulation containing 100 mg of vitamin B1, 200 mg of vitamin B6, and 200 µg of vitamin B12 (referred to as Brand A) was the most frequently recommended for patients presenting with numbness or sensory loss (52%) and those with overt DPN (41%). In contrast, a single-vitamin B12 product (Brand B) was primarily recommended for patients experiencing positive sensory symptoms, such as burning pain, tingling, lancinating pain, and pins and needles in the extremities (39%) (Figure 2).

Regarding symptom-specific recommendations, approximately 60% of pharmacists recommended combined neurotropic B vitamins for patients with both positive and negative symptoms. A smaller proportion recommended them exclusively for patients with positive symptoms (14%) or negative symptoms (6%). Notably, around 20% of pharmacists were uncertain about the symptom classification when making their recommendations (Table 3).

**Table 3.** Pharmacist practices regarding neurotropic B vitamins recommendation in DPN.

Characteristics		Overall (n = 90*)
Neurotropic B vitamins recommendation regarding symptom type, n (%)	Positive symptoms	13 (14%)
	Negative Symptoms	5 (6%)
	Both Positive and Negative symptoms	54 (60%)
	Not know of symptom types	18 (20%)
Treatment pattern of neurotropic B vitamins recommendation, n (%)	Monotherapy (neurotropic B vitamins only)	5 (6%)
	Adjunctive therapy (with standard treatment)	44 (49%)
	Both monotherapy and adjunctive therapy	41 (46%)
Treatment duration, n (%)	Until symptoms relieved; re-administer if symptoms return	15 (17%)
	On–off cycle (e.g. 3 months on, 1 month off)	32 (36%)
	Continuous treatment	41 (47%)
Dose selection factors, n (%)	Symptom severity	82 (91%)
	Clinical guideline recommendation	67 (74%)
	Patient characteristics (e.g. elderly, underlying diseases, concomitant medications)	63 (70%)
	Product information leaflet	63 (70%)
	Doctor recommendation	60 (67%)
	Patient's medication history	49 (54%)
	Other	1 (1%)
Treatment regimen, n (%)	Recommended dose as per product information leaflet, maintained throughout treatment course	28 (32%)
	Initiate high dose, reduce when improved	49 (56%)
	Initiate low dose, increase if poor clinical response	11 (13%)

* n = 90 pharmacists who recommended neurotropic B vitamins. DPN: diabetic peripheral neuropathy

The main treatment pattern of combined neurotropic B vitamins was used either as monotherapy or adjunctive therapy in nearly equal proportions (46–49%). Additionally, the pharmacist respondents recommended continuous treatment (47%) or on–off cyclical approach (e.g. 3 months on, 1 month off) (36%). The primary consideration for dose selection was symptom severity (91%) and clinical guideline recommendation (74%). A majority of pharmacists (56%) preferred initiating treatment with a high dose followed by dose reduction once symptoms improved. In contrast, only 13% reported initiating therapy with a low dose and escalating it if no clinical improvement was observed.

### Attitudes toward the pharmacist’s role in managing DPN and factors associated with recommending combined neurotropic B vitamins

**Figure 3.**
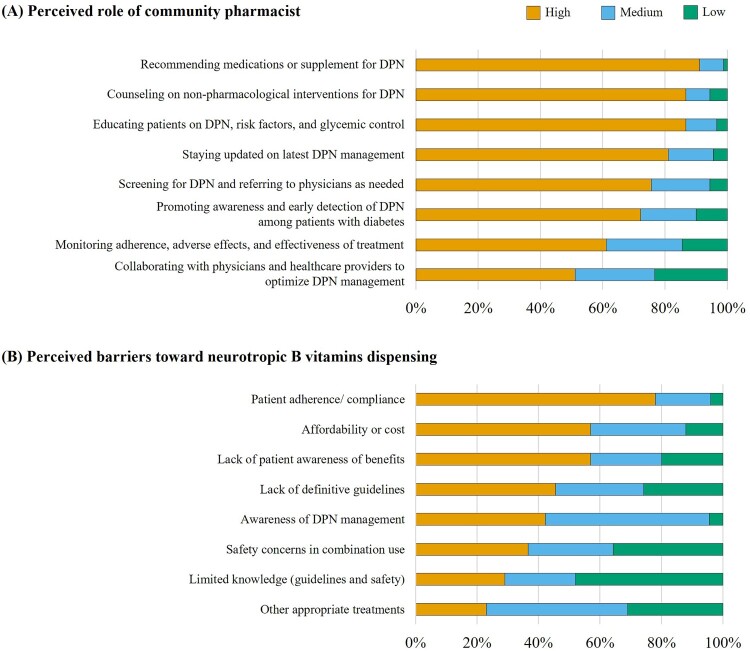
Perceived roles of community pharmacists and barriers toward recommending neurotropic B vitamins in diabetic peripheral neuropathy. The figure presents pharmacists’ perceptions regarding their roles and decision-making in the management of diabetic peripheral neuropathy (DPN). Panel (A) depicts the proportion of pharmacists endorsing specific professional roles, including medication recommendation, patient education, and non-pharmacological counselling. Panel (B) illustrates perceived barriers and factors influencing the recommending combined neurotropic B vitamins. Percentages represent the proportion of respondents selecting each option. Responses were based on pharmacists’ self-reported practices. DPN: diabetic peripheral neuropathy

Pharmacists’ perceived roles in DPN management primarily involved recommending medications or supplements (91%), providing counselling on non-pharmacological interventions (87%), and educating patients on DPN, its risk factors, and glycemic control (87%) (Figure 3A). Regarding perceived barriers and knowledge gaps, the top three factors influencing recommendation practices were concerns about patient adherence or compliance (78%), product affordability or cost (57%), and patients’ lack of awareness of treatment benefits (57%). In contrast, limited knowledge of clinical guidelines (48%) and concerns about the safety of combination use (36%) were less frequently cited as barriers to recommending combined neurotropic B vitamins (Figure 3B).

To further explore the decision-making process, multivariable logistic regression was used to assess the associations between product-related factors and the likelihood of pharmacists recommending combined neurotropic B vitamins (Table 4). Among patients presenting with negative symptoms, guideline endorsement was inversely associated with recommendation of combined neurotropic B vitamins (OR = 0.131; 95% CI: 0.027–0.626; *p* = 0.011), possibly reflecting the absence of painless DPN recommendations in most current clinical guidelines.

**Table 4.** Exploratory associations between product-related factors and pharmacists’ likelihood of recommending combined neurotropic B vitamins.

Factors	Negative symptoms		Positive symptoms		Overt DPN	
	OR (95% CI)	*p*-value	OR (95% CI)	*p*-value	OR (95% CI)	*p*-value
Mentioned in clinical guidelines	0.131 (0.027–0.626)	0.011*	0.486 (0.135–1.744)	0.268	0.629 (0.198–1.996)	0.431
Formulation contains multiple B vitamins	2.477 (0.352–17.430)	0.362	2.483 (0.284–21.715)	0.411	2.097 (0.393–11.179)	0.386
Vitamin doses within therapeutic range	0.497 (0.094–2.628)	0.411	0.168 (0.023–1.229)	0.079	0.274 (0.053–1.418)	0.123
Clear symptom indication on product labelling	0.946 (0.465–1.928)	0.879	0.370 (0.150–0.911)	0.031*	0.737 (0.358–1.516)	0.407
Well-known brand	0.587 (0.298–1.156)	0.123	0.586 (0.244–1.407)	0.232	0.467 (0.232–0.940)	0.033*
Reasonable price	1.267 (0.554–2.894)	0.575	3.993 (1.118–14.258)	0.033*	2.388 (0.943–6.045)	0.066

* Statistical significance, *p* < 0.05.

DPN: diabetic peripheral neuropathy, OR: odds ratio, CI: confidence interval.

A screenshot of a graph AI-generated content may be incorrect.

Regarding the positive symptoms, the presence of a clear symptom indication on the package labelling was also inversely associated with recommendation of combined neurotropic B vitamins (OR = 0.370; 95% CI, 0.150–0.911; *p* = 0.031). Reasonable product price was significantly linked to recommending combined neurotropic B vitamins (OR = 3.993; 95% CI, 1.118–14.258; *p* = 0.033). In addition, the brand reputation was conversely associated with recommending combined neurotropic B vitamins among overt DPN (OR = 0.467; 95% CI, 0.232–0.940; *p* = 0.033), possibly indicating a preference for lesser-known or cost-effective alternatives in community pharmacy practice.

## Discussion

The main findings of this exploratory survey study indicate that Thai community pharmacists frequently encounter patients with mild or early neuropathic symptoms and commonly recommend combined neurotropic B vitamins (100 mg of vitamin B1, 200 mg of vitamin B6, and 200 µg of vitamin B12) as first-line pharmacological therapy, particularly for patients presenting with negative sensory symptoms or overt DPN. Among product-related factors examined, only reasonable pricing showed positive association with the likelihood of recommending combined neurotropic B vitamins, whereas guideline endorsement, product labelling specificity, and brand recognition showed inverse associations. Given the cross-sectional design, convenience sampling, and self-reported data, these associations should be interpreted as exploratory signals warranting confirmation in larger studies rather than definitive determinants of prescribing behaviour. The observed patterns highlight important gaps between existing guideline-based recommendations and real-world pharmacy practice, especially in the management of painless or early-stage DPN.

DPN presents a significant clinical challenge, particularly in its early stages when intervention may prevent irreversible nerve damage. Many patients initially seek symptom relief from community pharmacies rather than physician consultation, as reflected in this study where approximately half of all presentations involved mild symptom severity. Community pharmacists assessed symptom severity primarily through clinical history-taking and patient-reported pain scales, with standardised screening instruments such as the DN4 questionnaire used infrequently (11–24% across symptom types). The predominance of mild presentations represents both an opportunity and a challenge: early pharmacist-led intervention may slow neuropathy progression, yet pharmacists often lack access to validated diagnostic tools and practice-specific clinical guidance (Sathienluckana et al., 2024). This gap is evidenced by the high proportion of treatment decisions involving medication refills or repetition of physician-initiated regimens, and the relative infrequency of non-pharmacological counselling despite pharmacists’ expressed willingness to provide comprehensive care.

In contrast to hospital-based settings, where specialist care and formal diagnostic protocols are routinely available, community pharmacists operate within distinct constraints that shape their DPN management approach. Hospital outpatient clinics employ validated diagnostic instruments such as the Michigan Neuropathy Screening Instrument and the DN4, enabling neurologists and endocrinologists to prescribe guideline-recommended first-line agents including duloxetine, pregabalin, or amitriptyline for painful DPN (Pop-Busui et al., 2022). However, a multinational survey in Southeast Asia revealed that even in hospital settings, DPN management faces barriers: limited consultation time, perceived inefficiencies, and a predominant focus on glycemic control, with fewer than 50% of physicians in Thailand, the Philippines, and Taiwan actively managing painful neuropathic symptoms (Malik et al., 2017). Community pharmacies, characterised by over-the-counter accessibility, patient self-referral, and predominantly mild or undiagnosed presentations, thus represent a critical but underserved point of care. The distinct practice environment underscores both the relevance of neurotropic B vitamins as a pragmatic first-line option and the urgent need for tailored clinical guidance (Sathienluckana et al., 2024).

Combined neurotropic B vitamins emerged as the most frequently recommended treatment for patients with negative symptoms (48%) and diagnosed DPN (45%), followed by single vitamin B12 and gabapentinoids. This preference aligns with the mechanistic role of B vitamins in nerve health: vitamin B1 supports energy metabolism and provides antioxidant effects, B6 facilitates neurotransmitter synthesis and nerve metabolism, and B12 promotes remyelination – collectively supporting nerve regeneration processes (Baltrusch, 2021). Recent in vitro studies demonstrate that combined B vitamin formulations significantly enhance neurite outgrowth, neural cell maturation, and connectivity compared to single B12 supplementation (Cuyubamba et al., 2025; Rayner et al., 2025), with systematic reviews supporting efficacy in improving neurophysiological outcomes in DPN patients (Farah & Yammine, 2022; Liew et al., 2019). The most frequently recommended product contained vitamins B1 100 mg, B6 200 mg, and B12 200 µg for twice- or thrice-daily dosing, placing total daily intake at or near the upper range of expert consensus recommendations (Sathienluckana et al., 2024; Schellack et al., 2025). In contrast, guideline-recommended agents such as tricyclic antidepressants, SNRIs, and gabapentinoids primarily provide symptomatic pain relief without modifying underlying neuropathy or supporting nerve regeneration (Mallick-Searle & Adler, 2024; Pop-Busui et al., 2022). However, potentially inappropriate use of paracetamol, NSAIDs, and gabapentinoids for negative symptoms was also observed, highlighting knowledge gaps that targeted education could address.

The observed inverse associations between guideline endorsement, package labelling clarity, and brand reputation with recommendation likelihood warrant careful interpretation within the Thai community pharmacy context. The inverse association with guideline endorsement, particularly pronounced for patients with negative symptoms, likely reflects a critical gap rather than non-adherence: existing clinical guidelines predominantly address painful DPN and provide minimal or no guidance for painless or early-stage presentations characterised by numbness and sensory loss (Mallick-Searle & Adler, 2024; Pop-Busui et al., 2022; Price et al., 2022). In the absence of explicit recommendations for these clinical scenarios, pharmacists appear to rely on clinical experience and perceived patient benefit rather than formal guideline direction.

Similarly, the inverse association with clear package labelling may be explained by regulatory constraints: many neurotropic B vitamin products in Thailand are labelled primarily for ‘numbness or sensory loss’ with limited reference to positive neuropathic symptoms such as burning pain or tingling. Consequently, pharmacists may hesitate to recommend products for symptom profiles not explicitly stated on packaging, even when emerging evidence suggests broader benefit. The inverse association with brand reputation, coupled with the positive association with affordable pricing, likely reflects practical realities of community pharmacy practice where cost-conscious patients and pharmacists may prefer effective, accessible alternatives over premium-priced branded products. These patterns collectively suggest that current regulatory frameworks and clinical guidelines inadequately address the real-world decision-making context of community pharmacists managing early or painless DPN. Clearer product labelling that explicitly includes positive neuropathic symptoms would likely facilitate more confident, evidence-based product recommendations across the full spectrum of DPN presentations.

Several limitations should be acknowledged. First, the cross-sectional design captures practices and perceptions at a single time point, precluding causal inference or assessment of changes in practice over time. Second, data were collected using self-reported questionnaires, which may be subject to recall bias and social desirability bias. Third, classification of positive and negative neuropathic symptoms relied primarily on patient-reported information and pharmacists’ interpretation rather than standardised diagnostic confirmation, introducing the possibility of symptom misclassification; while this reflects real-world practice, it may affect precision of symptom-specific treatment patterns. Fourth, the final sample size was substantially smaller than the calculated target, limiting statistical power and generalizability. The observed associations should therefore not be overstated as definitive determinants of recommendation behaviour but rather interpreted as preliminary signals warranting confirmation in adequately powered, representative samples. Finally, the use of online convenience sampling restricted to Bangkok may have introduced selection bias, as pharmacists who were more engaged or interested in DPN management may have been more likely to participate, further limiting generalizability to other Thai regions or countries. Despite these limitations, the study provides valuable preliminary insights into real-world community pharmacy DPN management and identifies important areas for future research and guideline development.

## Conclusion

This exploratory study among a convenience sample of Bangkok community pharmacists demonstrates active pharmacist engagement in early-stage DPN management, with combined neurotropic B vitamins (vitamin B1 100 mg, B6 200 mg, B12 200 µg) most frequently recommended for patients with negative sensory symptoms and overt DPN. Treatment decisions are driven primarily by symptom severity and product affordability. The inverse associations between guideline endorsement, product labelling, and brand reputation with recommendation behaviour likely reflect gaps in clinical guidance for painless neuropathy rather than inappropriate practice. Given the convenience sampling and limited sample size, findings should be considered preliminary. Pharmacy-specific clinical guidelines and clearer product labelling addressing both painful and painless DPN are warranted to optimise community pharmacy practice.
